# Should Pneumococcal Vaccines Eliminate Nasopharyngeal Colonization?

**DOI:** 10.1128/mBio.00545-16

**Published:** 2016-05-24

**Authors:** Larry S. McDaniel, Edwin Swiatlo

**Affiliations:** a Department of Microbiology and Immunology, University of Mississippi Medical Center, Jackson, Mississippi, USA; b Veterans Affairs Medical Center, Jackson, Mississippi, USA

## Abstract

*Streptococcus pneumoniae* remains an important human pathogen. For more than 100 years, there have been vaccine efforts to prevent pneumococcal infection. The pneumococcal conjugate vaccines have significantly reduced invasive disease. However, these vaccines have changed pneumococcal ecology within the human nasopharynx. We suggest that elimination of the pneumococcus from the human nasopharynx can have consequences that should be considered as the next generation of pneumococcal vaccines is developed.

## EDITORIAL

The primary goal of any vaccine is to prevent disease. Clearly human vaccines have been among the most significant developments in modern medicine. Prevention of a disease is more impactful than intervening once a disease has been established. The eradication of smallpox demonstrates the potential success that vaccines offer (1). However, human pathogens that are also part of the normal microbiota present a twist to the eradication paradigm, which has guided most vaccine development. Therefore, should the goal always be the complete elimination of the pathogen or maintaining a commensal state?

The conjugate vaccines have successfully reduced the incidence of pneumococcal disease. However, this success has led to a change in the epidemiology of pneumococcal disease. Thus, consideration should be given to alternative strategies for future vaccine development.

With the exception of ocular disease, it is well established that nasopharyngeal colonization precedes pneumococcal infection (2, 3). Typically, the pneumococcus maintains a commensal state when present within the human nasopharynx and has evolved factors that allow pneumococci to transiently exist in this environ. Most individuals who are colonized with the pneumococcus do not develop disease. Typically, a balance is maintained between host, microbiota, and the pneumococcus, a pathobiont (4) that can cause disease when this balance is disrupted. Additionally, when the pneumococcus enters a normally sterile body site such as the lungs, blood, or middle ear, some of the factors that allow it to exist as a commensal now contribute to the ability of the pneumococcus to cause disease.

It has long been held that the commensal microbiota serves to check the growth and spread of organisms that have pathogenic potential. These commensals accomplish this by effectively competing for scarce nutrients and suitable microenvironments. Additionally, there can be direct antagonism between bacterial species (4). This premise has provided impetus for the development of probiotics and microbial transplantation.

A current opinion is that if a pneumococcal vaccine can prevent colonization, then disease will be prevented. However, there are concerns that by eliminating a population of bacteria from its preferred niche, this may allow that void to be filled by pathogens that could cause similar diseases or potentially be more pathogenic. Additionally, perturbation of the microbial species that normally occupy a specific site in the body may lead to unintended consequences, such as disadvantageous alterations in the development of the immune system.

There are currently two licensed pneumococcal vaccines in the United States: the 23-valent pneumococcal polysaccharide vaccine (PPV23) and the 13-valent conjugate vaccine (PCV13). Both of these vaccines target disease-associated specific serotypes of the pneumococcal polysaccharide capsule. The capsule is the major virulence factor in invasive pneumococcal disease (IPD). PCV13 and its predecessor, PCV7, have significantly reduced IPD (5). PPV23 is a poor immunogen in children less than 2 years of age. Therefore, the PCV was developed as a pediatric vaccine with the aim of reducing IPD caused by antibiotic-nonsusceptible pneumococci among the very young (6). Additional serotypes were added to further reduce antimicrobial-resistant pediatric infections. Subsequently, the conjugate vaccine has been so successful at eliminating IPD causing serotypes among young children that PCV13 is now recommended for adults.

PPV23 was licensed for use in the United States in 1983. While there remains some question of the overall efficacy of PPV23, it remains part of a two-component immunization strategy, along with PCV13, for individuals with the highest risk, including immunosuppressed patients, such as those with HIV infection and the elderly (http://www.cdc.gov/vaccines/schedules/hcp/adult.html). Since the introduction of PPV23, there have been no changes in serotypes associated with colonization or IPD following the use of this vaccine. Why PPV23 fails to eliminate colonization by the covered serotypes is not fully understood. In contrast, the introduction of the PCV has changed pneumococcal ecology. While there has been a reduction in disease due to elimination of vaccine serotypes following the introduction of PCVs, there has been an increase in disease from non-vaccine serotypes and nonecapsulated *Streptococcus pneumoniae* (NESp). After the introduction of PCV7, antibiotic-resistant 19A strains emerged and were responsible, in part, for the reformulation to 13 serotypes. Since the deployment of PCV13, non-vaccine serotypes have continued to emerge. The most common serotypes depend on the population studied but include 6C, 8, 15A, 22F, and 23A,B (7–10).

As we march down the line of serotypes, eliminating carriage and invasive disease based on public health surveillance and academic reports, it is becoming evident that serotypes that did not previously appear on our radar are now increasingly identified in infections. Pneumococci are well adapted to the human nasopharynx, and elimination of a few serotypes from a human population only serves to open this niche to strains unaffected by host adaptive immune responses. It is obviously not reasonable to think we can induce immune responses directed at nearly 100 distinct polysaccharides simultaneously using current versions of conjugated vaccines.

There are some immediately obvious approaches to intervene in the complex relationship between pneumococci and humans. Ideally, a robust and long-lasting immune response that remains localized to the lower respiratory tract, blood, and lymphoid organs would protect against invasive disease while not disrupting the livelihood of the pneumococcus on the nasopharyngeal mucosa. We do not presently have the knowledge of what kind of immune response this might be, and such an immune response would not likely be effective for pneumococcal infections at mucosal surfaces, such as otitis media, sinusitis, and bronchitis. We may be able to prevent carriage and invasive disease by targeting antigens conserved across all serotypes, thereby reducing or eliminating pneumococcal burden on a rather impressively large scale. Many protein antigens have been suggested as vaccine candidates, and some have moved into human trials (11). Conserved proteins theoretically have the potential to absolutely eliminate pneumococci from the human population; however, pressure from immune responses may select for mutations that alter major epitopes or delete the gene altogether. Also, vaccines involving live attenuated pneumococci (12) or killed pneumococci (13) have been investigated. However, given the concerns regarding complex vaccines, the approval and general acceptance for these vaccines remain to be seen. Additionally, data from protein-based and whole-cell vaccine studies indicate that these vaccines may also eliminate the pneumococcus from the nasopharynx.

Alternatively, there may be some manipulations we can employ with the currently available polysaccharide-protein conjugation technology. Sequential immunizations at relatively short intervals with different vaccine formulations may allow us to cover all invasive serotypes in early childhood while minimizing exposure during nonimmune states. This would necessarily complicate an already crowded and complex childhood immunization schedule. Since there appears to be a niche in the human nasopharynx to which pneumococci are particularly adapted, it may be possible to fill this niche with pneumococci that are never invasive (if such exist). The lack of characterization of serotypes rarely isolated from clinical material and the propensity of pneumococci to take up and recombine exogenous DNA make this approach less appealing. With further characterization of the human respiratory microbiota, we may be able to manipulate the nasopharyngeal flora to create a less beneficial environment for pneumococci and reduce or eliminate carriage without the need for active immunization. Clearly, we have much to learn about control of immune responses with active immunization against pneumococci as well as the interaction of the taxa that inhabit the upper respiratory tract and make colonization possible.

There will be continued expansion in the use of the current pneumococcal vaccines that target the pneumococcal capsular polysaccharide. While there is ultimately a limit to the number of serotypes conjugated to a carrier protein that can be administered, we are likely to see more serotypes added to future versions of the PCV. However, the impact of elimination of specific serotypes from the pneumococcal population is not fully understood. It may be a more reasonable approach to develop vaccines that allow the pneumococcus to remain in the nasopharynx but prevent the dissemination to body sites that result in an infection.
